# Development and characterization of a eukaryotic expression system for human type II procollagen

**DOI:** 10.1186/s12896-015-0228-7

**Published:** 2015-12-15

**Authors:** Andrew Wieczorek, Naghmeh Rezaei, Clara K. Chan, Chuan Xu, Preety Panwar, Dieter Brömme, Erika F. Merschrod S., Nancy R. Forde

**Affiliations:** Department of Physics, Simon Fraser University, 8888 University Drive, Burnaby, BC V5A 1S6 Canada; Department of Chemistry, Memorial University, St. John’s, NL A1B 3X7 Canada; Faculty of Dentistry, University of British Columbia, Vancouver, BC V6T 1Z3 Canada; Department of Biochemistry, University of British Columbia, Vancouver, BC V6T 1Z3 Canada; Present Address: Department of Bioengineering, University of California at Los Angeles, Los Angeles, USA; Present Address: Green Innovative Technologies R&D Centre Ltd, Vancouver, Canada

**Keywords:** Collagen, Recombinant expression, HT1080 cells, Optical tweezers, Atomic force microscopy, Electron microscopy, Circular dichroism, Cathepsin K, Internal ribosomal entry site (IRES)

## Abstract

**Background:**

Triple helical collagens are the most abundant structural protein in vertebrates and are widely used as biomaterials for a variety of applications including drug delivery and cellular and tissue engineering. In these applications, the mechanics of this hierarchically structured protein play a key role, as does its chemical composition. To facilitate investigation into how gene mutations of collagen lead to disease as well as the rational development of tunable mechanical and chemical properties of this full-length protein, production of recombinant expressed protein is required.

**Results:**

Here, we present a human type II procollagen expression system that produces full-length procollagen utilizing a previously characterized human fibrosarcoma cell line for production. The system exploits a non-covalently linked fluorescence readout for gene expression to facilitate screening of cell lines. Biochemical and biophysical characterization of the secreted, purified protein are used to demonstrate the proper formation and function of the protein. Assays to demonstrate fidelity include proteolytic digestion, mass spectrometric sequence and posttranslational composition analysis, circular dichroism spectroscopy, single-molecule stretching with optical tweezers, atomic-force microscopy imaging of fibril assembly, and transmission electron microscopy imaging of self-assembled fibrils.

**Conclusions:**

Using a mammalian expression system, we produced full-length recombinant human type II procollagen. The integrity of the collagen preparation was verified by various structural and degradation assays. This system provides a platform from which to explore new directions in collagen manipulation.

**Electronic supplementary material:**

The online version of this article (doi:10.1186/s12896-015-0228-7) contains supplementary material, which is available to authorized users.

## Background

Collagens are the fundamental structural proteins in vertebrates, where they fulfill a variety of critical roles in connective tissue structure and mechanics. As such, alterations in collagens’ composition, resulting from genetic modifications, aging, and diabetes, have been identified with an extensive list of diseases [1, 2]. Additionally, due to their natural role as the structural component in the extracellular matrix, collagens have found widespread use in biomaterials, used for cellular and tissue engineering, drug delivery, and a wide range of other applications [3–5].

Most studies on collagens use protein extracted from animal tissues. While this provides a large-scale supply of the protein, the lack of control over protein composition has its drawbacks. For example, there is minimal ability to select protein sequence, since generally type I collagen is most easy to extract and its sequence varies little among different animal species. Furthermore, because posttranslational modifications play a role in collagen’s mechanics, and can influence cellular phenotype, batch-to-batch variability in collagen composition can arise due to animal age or diet [6–10]. To surmount issues arising from variability of tissue-derived collagen, an alternative strategy employs harvesting collagen directly from cultured cells. A benefit of this approach is the ability to gain insight into the etiology of disease by using patient-derived cells. However, because most collagenopathies are heterozygous, harvesting collagen from these cell lines results in a mixture of both wild-type and mutant proteins.

To overcome these challenges and exert control over collagen’s sequence, recombinant expression systems have been developed. These utilize a host cell line to express the desired collagen gene of interest, permitting expression of mutated genes and also of completely novel protein sequences. Benefits of a recombinant expression system include control over the expressed protein sequence, control over extent of posttranslational modifications, and reproducibility of culturing conditions and hence protein composition [11–16]. Because collagen is harvested shortly after expression, it is also devoid of age-related crosslinks inherent to tissue-derived samples, thus having the potential to serve as an ideal source of “young” collagen for studies on aging. The ability to alter protein composition in a controlled manner suggests the opportunity to engage in rational design of materials, by correlating composition of the collagen building blocks with desired mechanical properties of self-assembled structures, offering the potential of tuning parameters such as fibril diameter and pore size within a matrix via protein composition.

To date, collagen has been expressed in a variety of host cell lines [4, 15, 17–26]. Because fibrillar collagens require posttranslational modifications such as proline hydroxylation for stable folding of the triple helix, this constraint must be accommodated in any recombinant expression system. Thus, while bacteria generally offer easy access to protein expression, their lack of endogenous posttranslational machinery makes the expression of stable triple helical collagen challenging, requiring co-expression of enzymes such as prolyl hydroxylase [15, 19, 21, 22]. More success has been obtained in yeast lines, again by co-expressing prolyl hydroxylase, which have produced full-length protein with a thermal stability similar to that of wild-type and have been used as a viable source of collagen at industrial levels [4, 19]. The successful use of this collagen in tissue implants demonstrates the feasibility of using recombinant human collagen for in vivo biomaterials applications [27–29]. However, this expression system does not encode for the numerous other posttranslational modifications, such as hydroxylation of lysines and glycosylation of the hydroxylysines, that are part of collagen’s higher-order assembly pathway and affect its stability and physiological function [6, 13]. To encode each of these additional enzymatic modifications would add yet more complexity to the expression system, requiring additional genetic manipulation for each added post-translational modification. A more direct route to fully modified collagen is preferred.

For applications seeking a more realistic model of disease, cells possessing and expressing the full suite of posttranslational modification machinery are required. Mammalian cells possess all of the genetic instructions to do so. Earlier work demonstrated that the HT1080 fibrosarcoma cell line endogenously expresses this suite of enzymes, producing correctly modified collagen from a recombinant expression system [17]. This system has enabled studies of sequence-dependent structural changes of triple helical type II collagen monomers and of morphological changes of self-assembled fibrils [30–32]. We wished to exploit the success of this work, and to develop a similar system for collagen expression that would enable more facile screening for stable protein expression. To that end, we have developed a recombinant expression system for type II procollagen in this previously validated HT1080 cell line.

Type II collagen is the second-most abundant fibrillar collagen and is found in cartilage, the vitreous humour of the eye, the inner ear, and in intervertebral disks. It is the predominant protein component of articular cartilage, whose enhanced digestion is associated with aging and is particularly severe in osteo- and rheumatoid arthritis [33, 34]. Mutations in the COL2A1 gene encoding type II procollagen can lead to diseases including achondrogenesis, hypochondrogenesis and various skeletal dysplasias [35]. Type II collagen matrices have been used to support cell growth and have proven particularly useful for promoting proliferation of chondrocytes, which are important for repair of damaged cartilage [28, 29, 36–38].

**Fig. 1:**
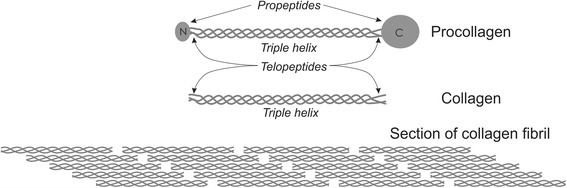
Schematic of procollagen and collagen structures. Procollagen is purified from the cell culture medium. Post-purification enzymatic processing results in removal of the propeptides, creating a form of collagen (consisting of both triple helix and telopeptide regions) capable of self-assembly into fibrils. A portion of a collagen fibril, illustrating highly ordered lateral packing (*D-banding*), is shown

Here, we describe a human type II procollagen recombinant expression system that utilizes a fluorescent marker to screen for selection of stably transfected human fibrosarcoma cells that produce endogenously post-translationally modified protein [39]. Though inspired by a closely related system [17], ours differs in that it expresses the complete sequence of wild-type procollagen and utilizes a fluorescence-based reporter system for monitoring expression, thereby facilitating confirmation of stable expression. Notably, the fluorescence reporter is co-expressed with the procollagen but is not fused to it, differing from other expression systems [40]. This approach avoids possible disruption of folding, assembly or secretion of the native form of the protein and to our knowledge has not been applied previously to collagen production. In our system, the procollagen is produced as an isolated full-length protein in its native form, permitting facile comparison with procollagen purified from patient-derived cell lines. Thorough biochemical and biophysical characterization of the purified protein demonstrates that this easy-to-screen recombinant expression system produces properly structured and biochemically recognized collagen at the molecular level, capable of self-assembly into fibrils (Fig. 1). The demonstrated fidelity of the system opens the doors to the use of this recombinantly produced protein in a wide variety of fundamental and applied assays, offering tunable control over molecular parameters not accessible in tissue-derived samples.

## Results and discussion

To produce post-translationally modified type II human procollagen, HT1080 human fibrosarcoma cells were used as the host cell line. This cell line was chosen for the transfection and expression of the recombinant protein because its endogenous expression of collagen IV provides the requisite enzymes for correct post-translational modification and secretion of the recombinant type II procollagen [17].

**Fig. 2:**
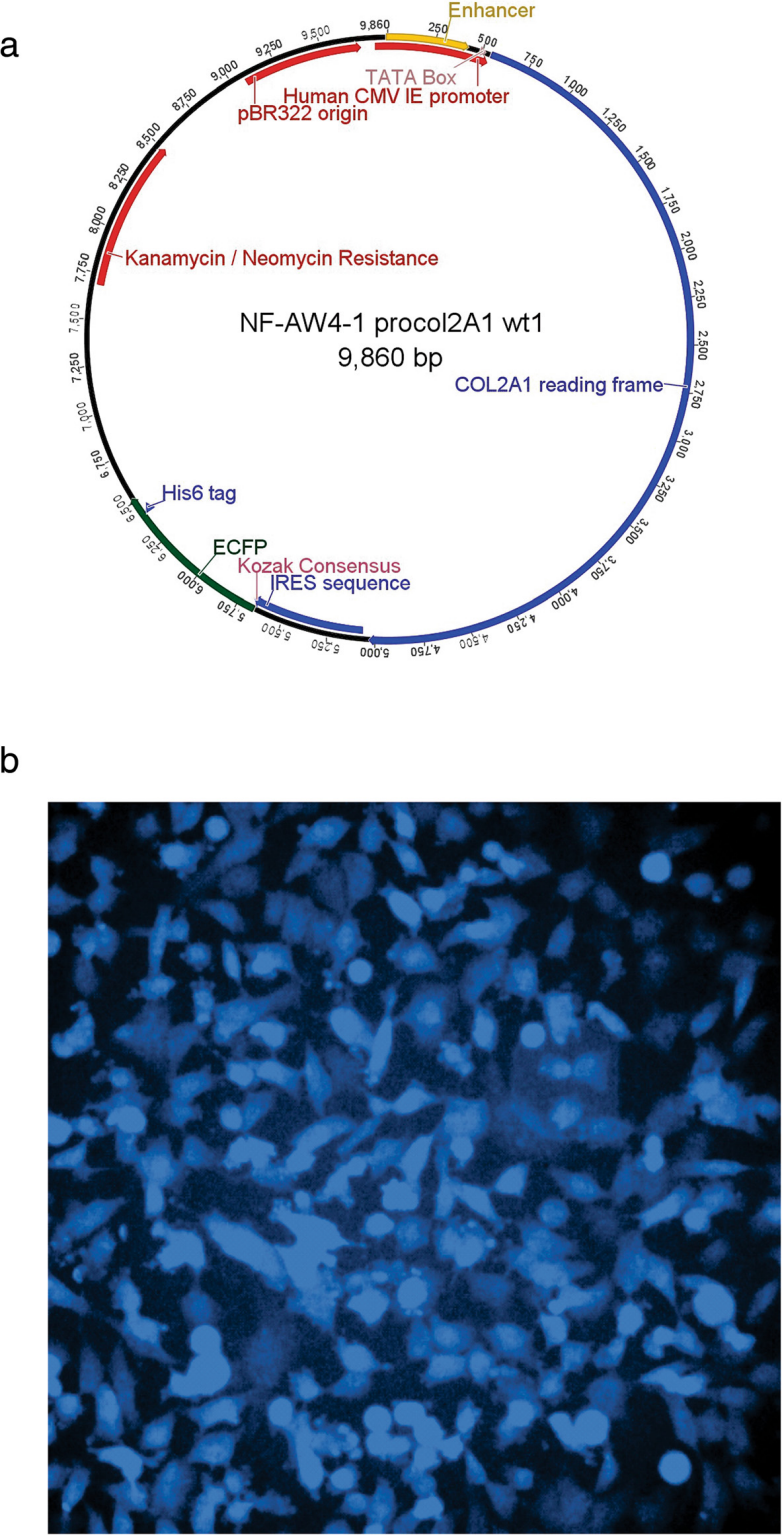
Expression of recombinant human type II procollagen. **a** Expression vector transformed into HT1080 cells, showing location of the COL2A1 procollagen gene, the IRES sequence and the ECFP gene. **b** Confocal fluorescence microscopy image of HT1080 cells stably transfected with COL2A1; the blue color results from co-expression of ECFP

We sought an expression vector that produced an easy screening mechanism for selection. The pYIC vector (Addgene) was chosen, as it incorporates an aminoglycosidase which allows for selection in both bacterial (kanamycin) and eukaryotic (G418) systems. In this vector, we replaced the gene for enhanced yellow fluorescent protein (EYFP) with that of cDNA-derived human type II procollagen (IMAGE Consortium, [41]). This resulted in the plasmid shown in Fig. 2a. Following transfection into HT1080 cells, this construct gave rise to simultaneous, uncoupled translation of procollagen and a downstream marker protein used to screen the cells, enhanced cyan fluorescent protein (ECFP), from a single mRNA transcript using an internal ribosome entry site (IRES) located between the two open reading frames. The blue ECFP fluorescence from the transformed cells is an indirect, but coupled, indicator of the expression of procollagen and was used to screen the cells. By performing serial dilution and subsequent expansion of transfected cells, we obtained a uniform stably transfected population expressing procollagen, as seen by the blue fluorescence signal from all cells in Fig. 2b.

**Fig. 3:**
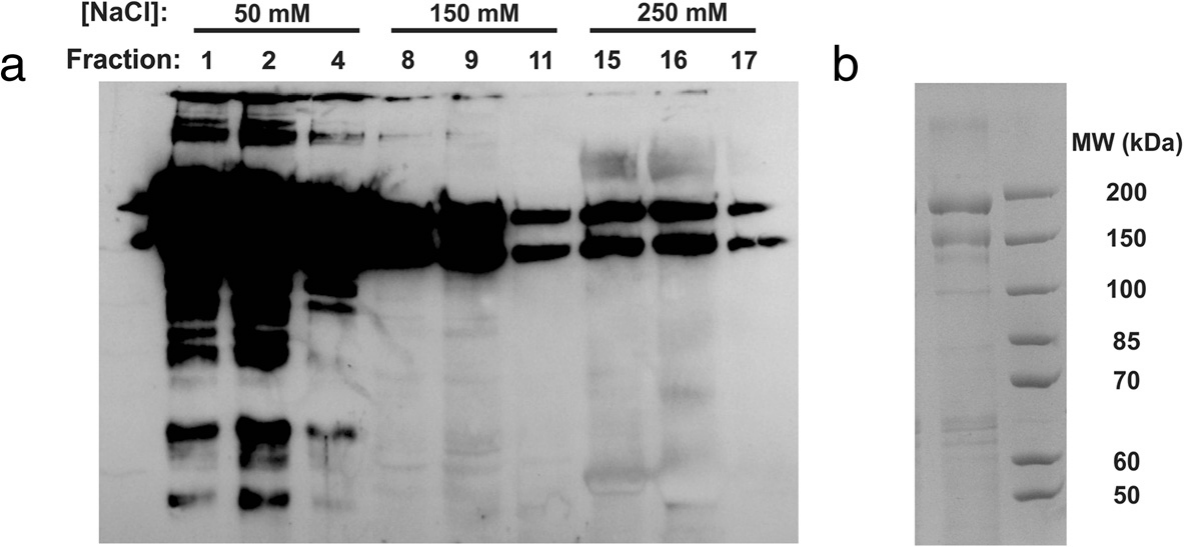
FPLC purification of type II human recombinant procollagen from HT1080 cell line. **a** Western blot for type II collagen of samples eluting from the Q-sepharose column. Samples were eluted in Q sepharose buffer plus a step gradient of NaCl as indicated. Numbers at the top the lanes refer to the fraction collected, and samples are loaded in equal volumes into each lane of the gel. The earliest fractions contain the most procollagen; this decreases with increasing ionic strength. **b** Coomassie-stained gel showing pooled fractions 1–4 (*left lane*) and a molecular weight marker (*right lane*). The two bands of highest molecular weights are full-length type II procollagen pro-α chains, presumably with different internal crosslinking in the propeptides (see text)

Type II procollagen was purified from the cell media by modifying a literature-based protocol [17] as described in the methods section. The peak elution from the Q-Sepharose anion-exchange column occurred at low NaCl (Fig. 3a). Bands corresponding to the purified protein are shown in the gel of Fig. 3b. Eluted fractions displaying strong collagen signal were pooled and concentrations were assessed using the Sircol assay [42], which has high sensitivity for triple helical collagen. Typical final concentrations were 80 μg/ml, though could range up to 150 μg/ml. Each harvest yielded 10-12 ml of this purified collagen, for a total yield of ~1 mg procollagen per liter of medium. In order to boost this yield, strategies to increase cellular density during culturing, such as the use of suspended microcarriers or fixed-bed reactors, could be considered.

Coomassie-stained gels show the predominant presence of high-molecular-weight species, demonstrating the purity of our sample (Fig. 3b). We observe two bands in the vicinity of the expected molecular weight (142 kDa for full-length procollagen); this observation of two bands in a purified sample has been seen previously for type II procollagen [30]. Both high-molecular-weight bands are recognized by an antibody specific to the N-telopeptide sequence of type II collagen that does not cross-react with other collagen types (Fig. 3a). As discussed below, the purified protein collapses to a single band following chymotrypsin treatment to remove the propeptides, i.e., these mobility differences do not reflect differences within the triple helical collagen structure.

To provide further evidence of the identity of the purified protein, and to check for expected posttranslational modifications, protein analysis (tandem mass spectrometry (MS/MS) identification of tryptic fragments, UVic-Genome BC Proteomics Centre) was performed. A search of the identified peptides against the Uniprot-Swissprot database found the highest match to be with human type II procollagen, with a MOlecular Weight SEarch (MOWSE) score of 3666 [43]. Sequence coverage of identified tryptic peptides represented 62 % of this large protein (Additional file 1: Figure S1). Peptide mass analysis showed expected post-translational modifications of hydroxyproline, hydroxylysine, galatosyl-hydroxylysine and glucosyl-galactosyl-hydroxylysine (Additional file 1: Figure S1). This provides evidence of the fidelity of expression and purification of post-translationally modified human type II procollagen from our system.

**Fig. 4:**
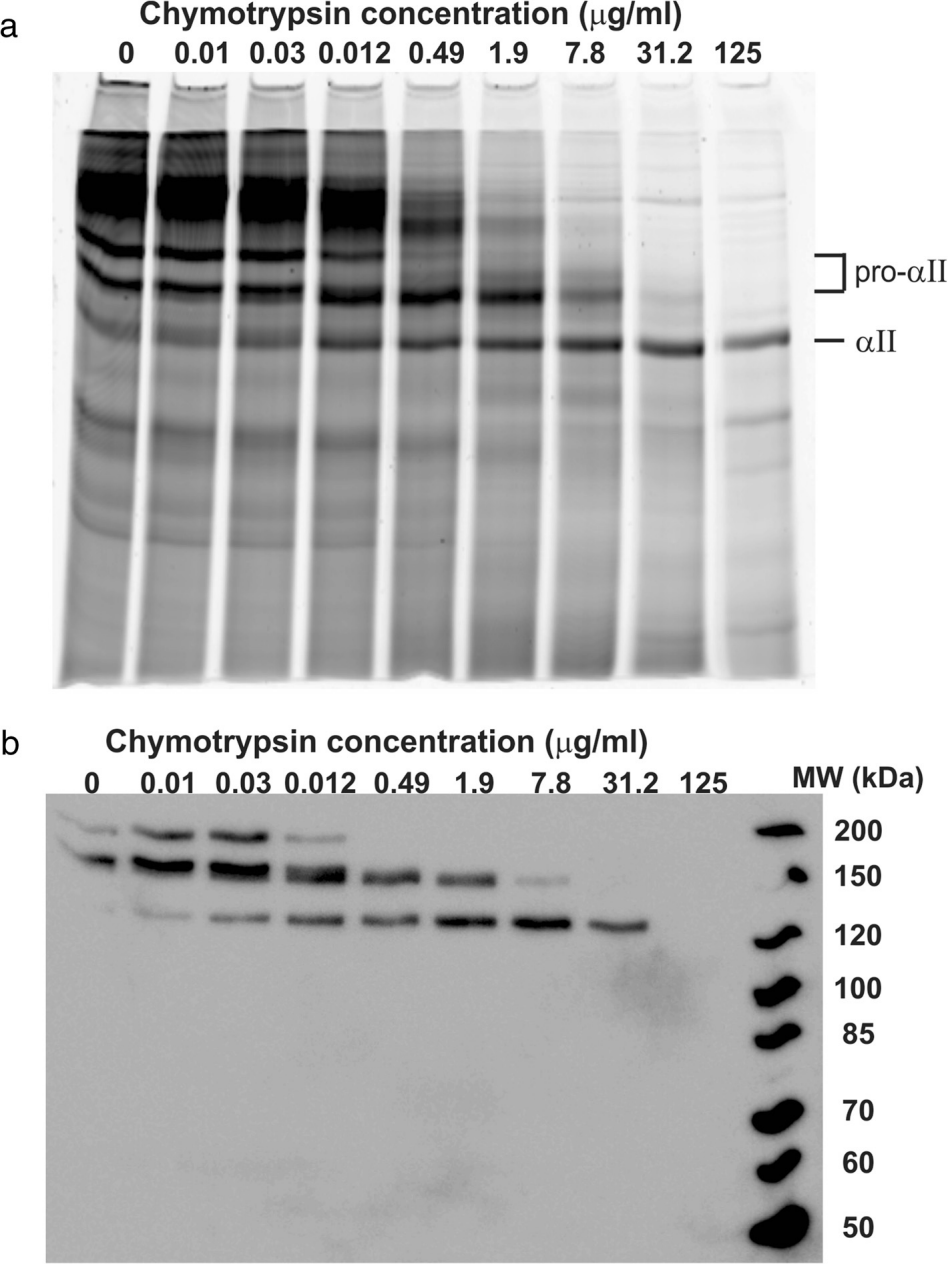
Chymotrypsin digest of recombinant type II human procollagen. Alexa 647-labelled procollagen was incubated with different concentrations of chymotrypsin for 30 min at 4 °C. Increasing concentrations led to successful removal of the propeptides, while leaving the triple helix intact, as evidenced by the collapse of all signal into a unique, high-MW band following incubation with 31.2 μg/ml chymotrypsin. **a** Fluorescence scan of the gel, showing all protein in the sample. **b** Western blot with a monoclonal antibody to the N-telopeptide. This Western shows that the high-MW signal is due to collagen, and furthermore demonstrates that only at the highest concentration is the telopeptide epitope removed

**Fig. 5:**
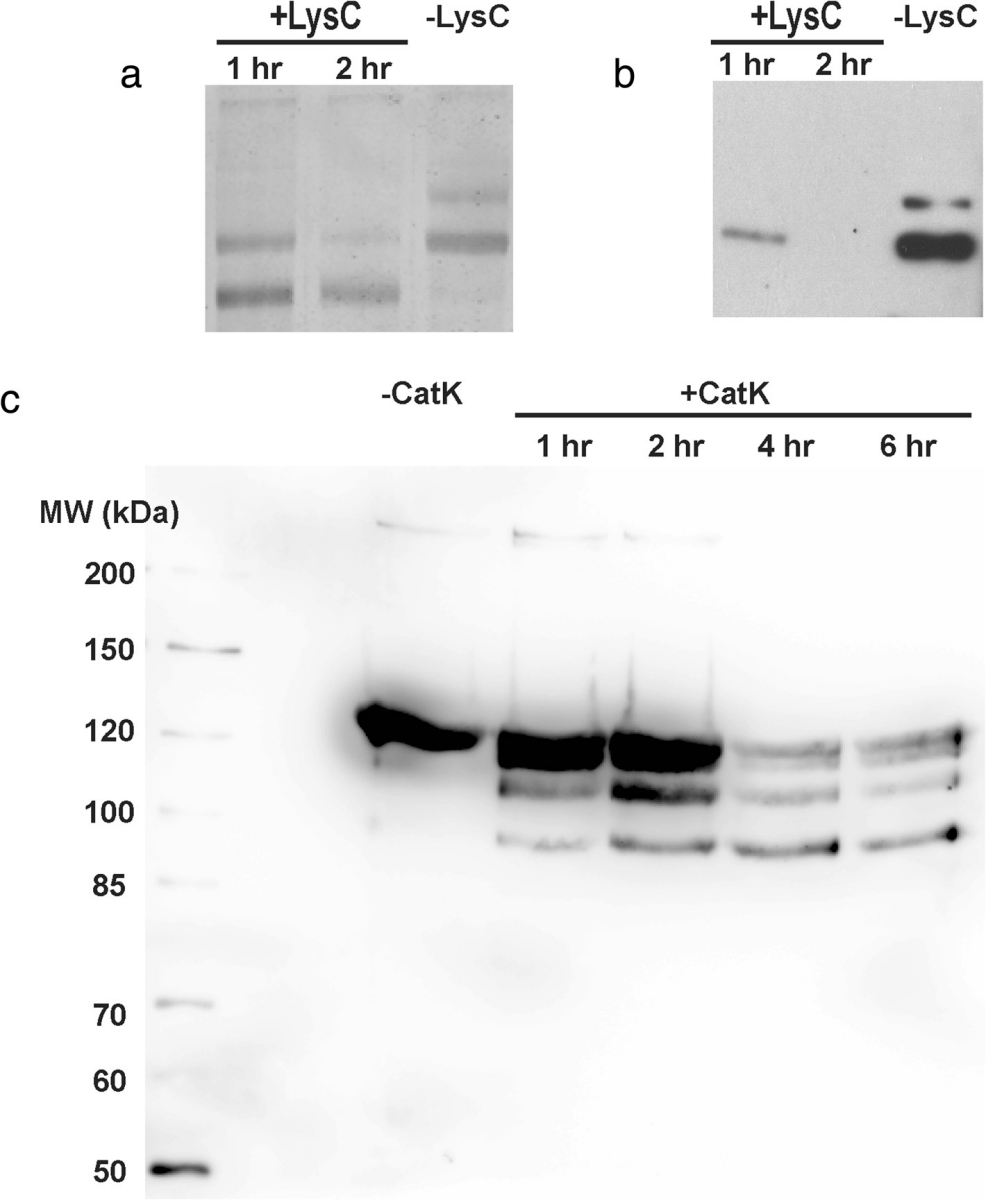
Proteolytic digestion by Lys-C or cathepsin K shows expected cleavage pattern. **a** Lys-C incubation with purified type II procollagen shows a reduction in protein size, as seen by silver staining, consistent with removal of N- and C-propeptides. **b** Western blot with an antibody specific to the N-telopeptide shows that shorter incubation times result in the removal of propeptide but not telopeptides, while longer incubations result in cleavage of the N-telopeptide by Lys-C. **c** Western blot showing increasing time-dependent cleavage of type II collagen (prepared by chymotrypsin digestion of procollagen) by recombinant cathepsin K

We wished to confirm that the purified protein was correctly assembled into a triple helical structure. To do so, protease digestion was used as an initial assay, as the triple helix of collagen is resistant to digestion by most proteases [44]. The purified procollagen was incubated with different concentrations of chymotrypsin for 30 min at room temperature (Fig. 4). An increase in protease concentration resulted in a greater extent of digestion of procollagen, but even at the highest concentrations used, a single high molecular-weight (MW) band remained in the gel, correlating with the presence of the intact collagen triple helix. (Corresponding with collagen’s known anomalous mobility, its 95 kDa band runs more slowly than the standards [45]). At the highest concentration of chymotrypsin the (non-triple-helical) N-terminal telopeptide of collagen was removed, as indicated by the disappearance of the high-MW band in the Western using an antibody targeting this epitope, though the triple helix remained intact. A similar shift from procollagen to collagen was observed following treatment of the purified protein with lysyl endopeptidase (Lys-C) (Fig. 5) [32]. The lack of degradation of the α-chains of the core region of collagen following treatment with either of these proteases is evidence of the stability of its extended triple helix.

**Fig. 6:**
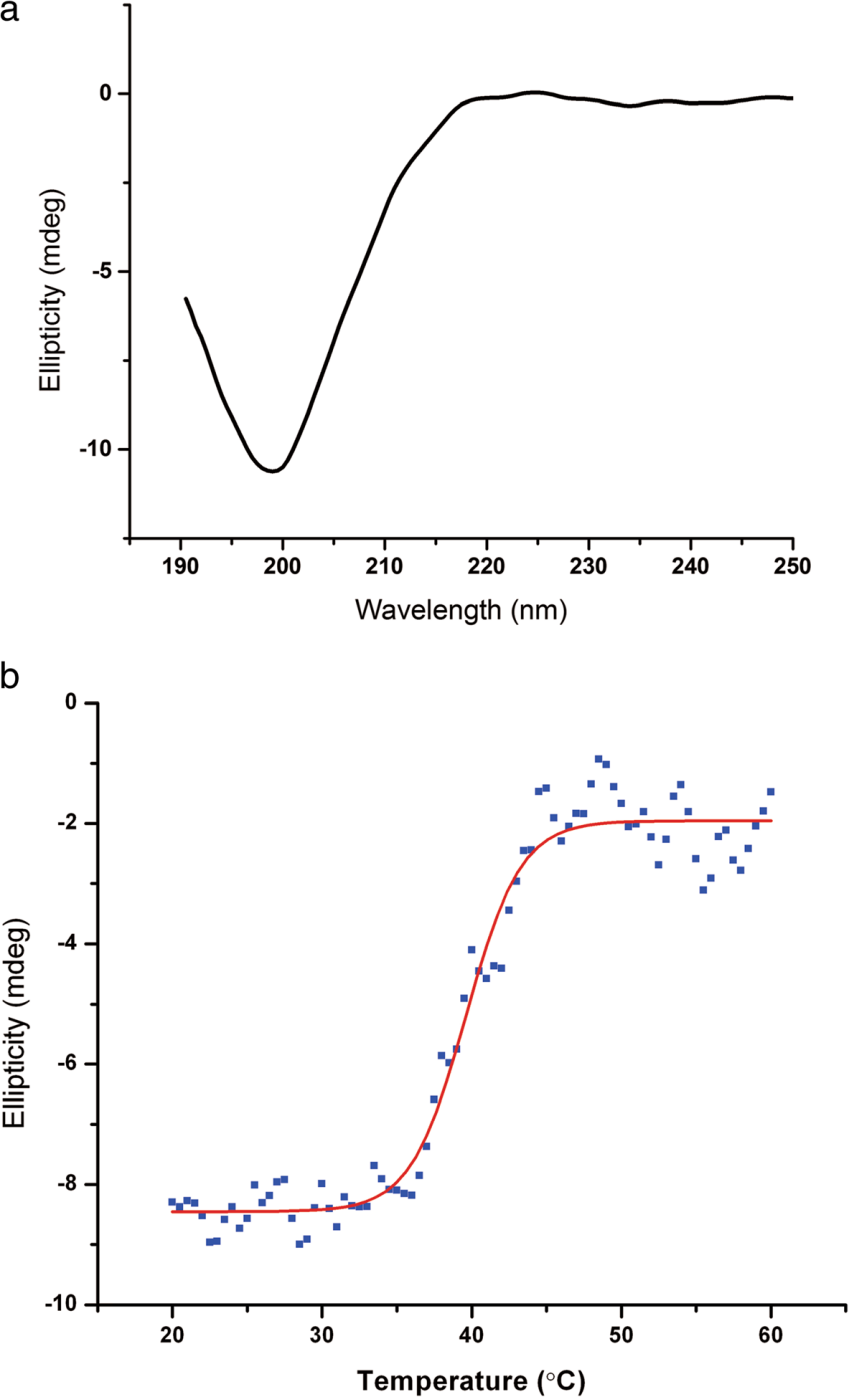
Circular dichroism (CD) spectroscopy to probe collagen’s triple helical structure. **a** CD spectrum of our type II collagen, produced by Lys-C digestion of recombinant human type II procollagen, shows significant negative ellipticity at 198 nm and a slight peak at 223 nm, indicative of proper formation of the triple helix. **b** Thermal melt curve for the type II collagen sample of (a), measured by recording the ellipticity at 198 nm as a function of temperature. The temperature was increased at a rate of 0.4 °C/min. As the triple helix denatures, ellipticity is lost at 198 nm. The melting temperature obtained from a fit to this plot with equation (2) (red line) is T_m_ = 39.6 °C

To assess the thermal stability of the triple helix, we measured the melting temperature using circular dichroism (CD) spectroscopy. Here, we used Lys-C-generated collagen to eliminate any influence of propeptides on the results. The CD spectra showed the expected shape for triple helical collagen, displaying significant negative ellipticity at 198 nm and a slight peak at 223 nm (Fig. 6a). By measuring the change in CD as a function of temperature, we showed that collagen thermally denatured near the expected 37 °C (Fig. 6b) [31, 46, 47]. A fit to the denaturation curve using equation (1) gave a melting temperature of *T*_m_ = 39.6 °C. As is well established for collagen, its irreversible nature of unfolding results in an overestimate of the true melting temperature for the scan speeds used here, [47] and this value for *T*_m_ is similar to values previously reported using this technique [31].

As a further assessment of the correspondence of our recombinant type II collagen to the native version, we examined its cleavage pattern when treated with the collagenase cathepsin K [48]. We found that cathepsin K cleaves recombinant type II collagen (Fig. 5c), giving a banding pattern upon enzymatic digestion consistent with previous findings on tissue-derived type II collagen [48, 49]. Furthermore, the time-dependent appearance of the discrete cleavage bands also agrees with results on tissue-derived type II collagen [48, 49].

**Fig. 7:**
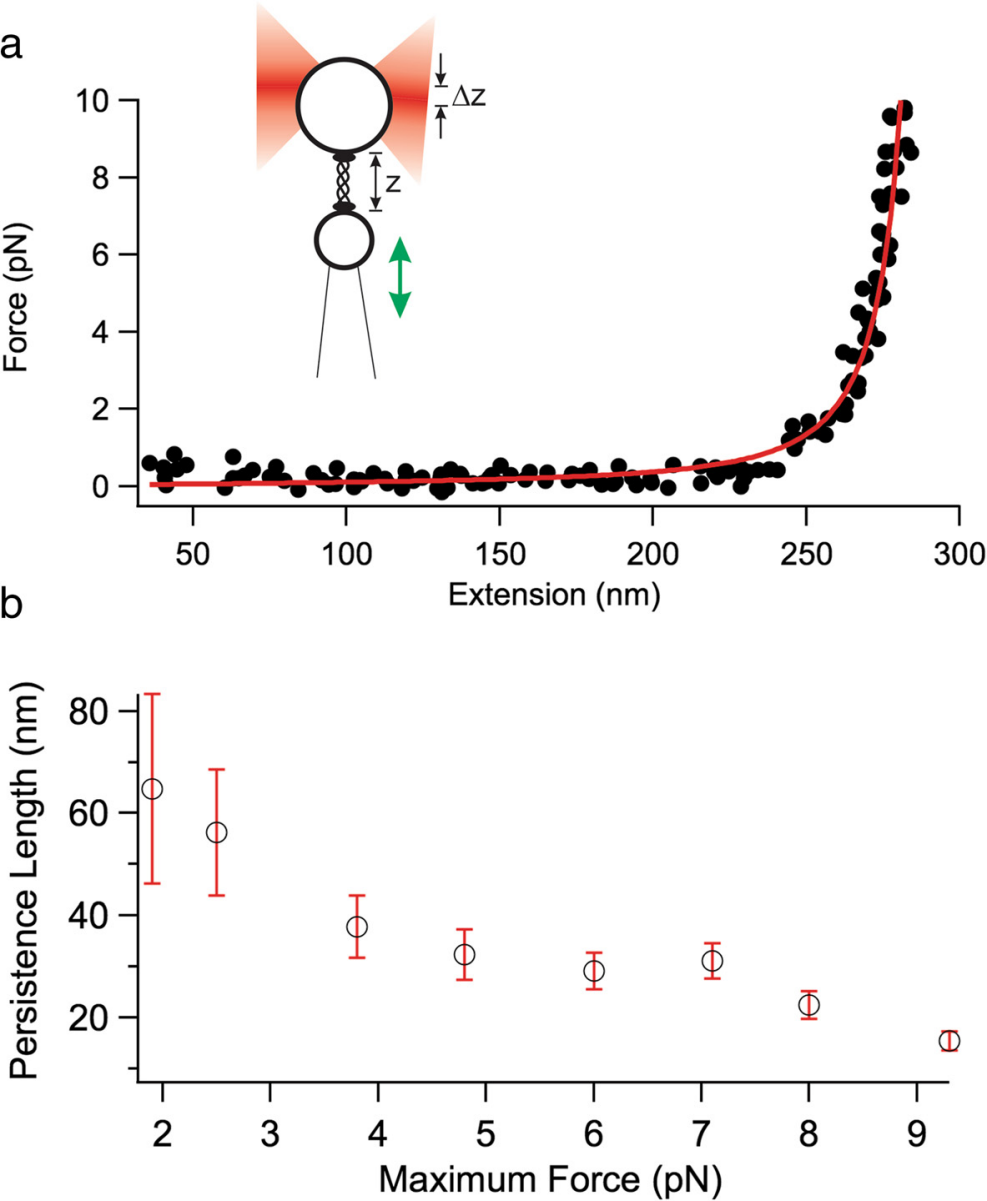
Optical tweezers stretching curves of type II procollagen described at low force by entropic elasticity. **a** The Worm-Like Chain (WLC) model (red; equation (3)) is fit to an example force-extension curve (black dots), giving a persistence length of 32 nm for a molecule of 300 nm contour length, when a maximum force of 5 pN is used for the fit. Inset: a schematic showing procollagen stretching in the optical tweezers and illustrating the extension *z* and bead offset from trap Δ*z*, from which force is determined. Schematic is not to scale. **b** The persistence length from fitting the WLC model decreases as the maximum force used in the fitting increases. The error bars show the uncertainty of the fitting parameter

A final assay at the molecular level employed optical tweezers to stretch single molecules of our recombinant type II procollagen. The resulting force-extension curves were analyzed, first to ensure that they corresponded to a single molecule, and then to extract information on molecular flexibility. Previous optical tweezers studies investigated the force-extension behavior of types I and II procollagen, freshly obtained from mammalian cells in culture [50, 51]. There, collagen was described as possessing entropic elasticity at forces *F* < 10 pN, i.e., that stretching collagen at these low forces removes configurational entropy but does not deform native structure. This intrinsic flexibility of triple-helical collagen was described by the persistence length, a parameter that describes the length scale over which a polymer can be thought of as unbent (rigid). The force-extension behavior we observed for our type II procollagen can similarly be fit at low forces by the inextensible worm-like chain model (equation (3)), as seen in Fig. 7.

Analysis of an example curve demonstrates the sensitivity of the output persistence length to the range of forces included in the fit. While fitting the data up to a maximum force of ~10 pN returned a persistence length comparable to values previously published in the literature, limiting the data range to lower maximum forces resulted in a systematic increase in the best-fit persistence length (Fig. 7b). This result has not been observed before for single collagen molecules. While persistence length is sensitive to parameters such as slight geometric offsets between the tethering and stretching axes, [52] it is possible that the systematic trend observed here reflects a force-dependent structural transition that could alter the stability of the triple helix as it is stretched [53–55]. Characterization of the force dependence of collagen’s structure is beyond the scope of the current work; here the agreement in persistence length within a similar force range used by previous optical tweezers studies adds further evidence to the proper assembly of collagen at the molecular level.

In its physiologically abundant form, collagen is found not as isolated molecules but incorporated into fibrils. Thus, we wished to verify that our recombinant collagen was capable of fibril assembly and to characterize this process and the properties of the assembled fibrils. These experiments necessitate removal of propeptides to enable fibril assembly (Fig. 1), and so, to generate a form of collagen capable of fibril formation, we cleaved procollagen II with Lys-C (Fig. 5) [32]. The cleavage sites of Lys-C lie 9-10 residues internal to the cleavage sites of the endogenous N- and C-terminal propeptidases, but this slightly truncated collagen nonetheless has been shown previously to produce fibrils morphologically indistinguishable from those prepared from the full-length collagen [32].

**Fig. 8:**
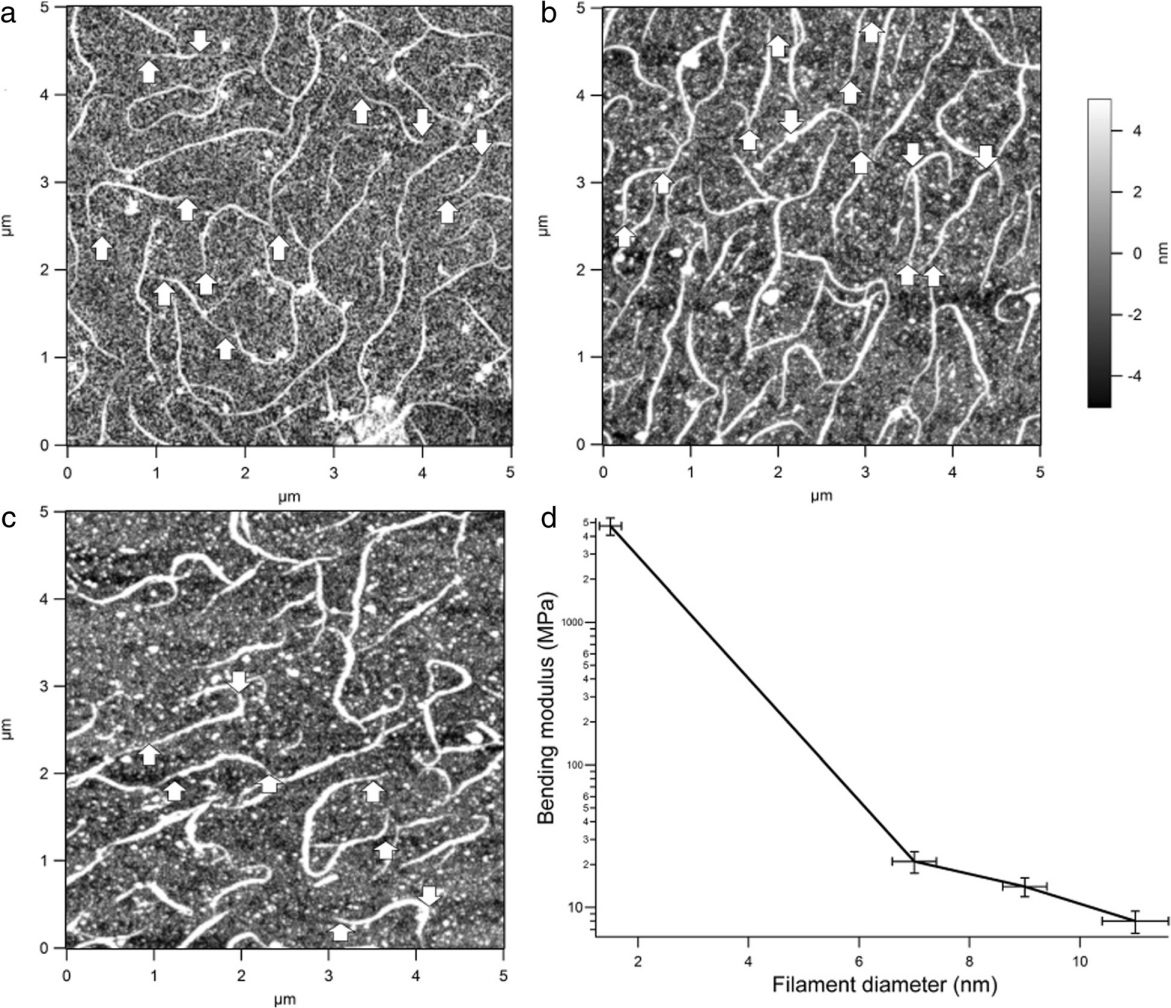
Atomic force microscopy analysis of type II collagen fibrillogenesis. **a**-**c** Images of collagen fibrils formed after **a** 10 min, **b** 20 min, and **c** 30 min of incubation. The upward pointing arrows show tapered ends and downward pointing arrows show blunt ends. **d** Bending modulus versus filament diameter extracted from AFM images at different time points of the fibrillogenesis process

Fibrillogensis of the Lys-C treated type II collagen sample was characterized by atomic force microscopy (AFM) imaging (Fig. 8) [56, 57]. After 10 min, filaments grew to 1–3 μm long and around 8 nm high (Fig. 8a). One can observe asymmetric morphologies in the shorter (less than 1.5 μm long) filaments, with one tapered and one blunt end, suggesting a unipolar structure [58, 59]. Both ends of longer filaments tend to appear tapered, indicating that in some cases fibril growth continues from both ends. After 20 min, the fibril height increases to around 9 nm (Fig. 8b), but without a corresponding increase in length. After 30 min, the fibril height increases to around 10 nm and their length appears unchanged (Fig. 8c). No significant change can be observed under further incubation of up to 24 h. Therefore, when grown under these conditions, the fibrils become mature after 30 min of incubation. As before, both unipolar and bipolar fibrils are observed.

From these images, the bending modulus of fibrils at different stages of assembly was extracted. Equation (4) was used to determine persistence lengths from angular correlations along the collagen fibrils. From this value and the height (diameter) [60] of the fibrils, the bending modulus is given by equation (5). This approach to extracting mechanical parameters has been applied to other types of images as well [61, 62]. As the method does not require indentation, pulling, or other direct manipulation of the sample it offers advantages in measuring soft and thin samples [63, 64]. The link between persistence length and mechanical properties is well established [62], including direct comparative measurements of mechanical response from persistence length and from stretching [65]. Our analysis assumes the collagen samples to be equilibrated on the surface prior to drying (two-dimensional equilibration). If they are instead two-dimensional projections of solution conformations, or pinned somewhere between the two-dimensional and three-dimensional cases, then estimates for persistence length and hence bending modulus will be significantly different [66, 67].

A plot of bending modulus versus filament diameter is shown in Fig. 8d, which also includes the data for the earliest stages of formation. These data indicate that the bending modulus decreases as fibril diameter increases, with a bending modulus for the thickest 11 nm diameter fibrils of around 8 MPa. While the persistence length should depend on the diameter, as seen in equation (5), the bending modulus is not presented as depending on diameter. In fact, however, the bending modulus does change with diameter. This decrease in stiffness for fibrils *vis a vis* monomers has been observed for type I collagen and can be explained by the weaker interactions between components in a fibril (monomer-monomer interactions) than between components in a monomer (a triple helix held together by many hydrogen bonds [68]).

**Fig. 9:**
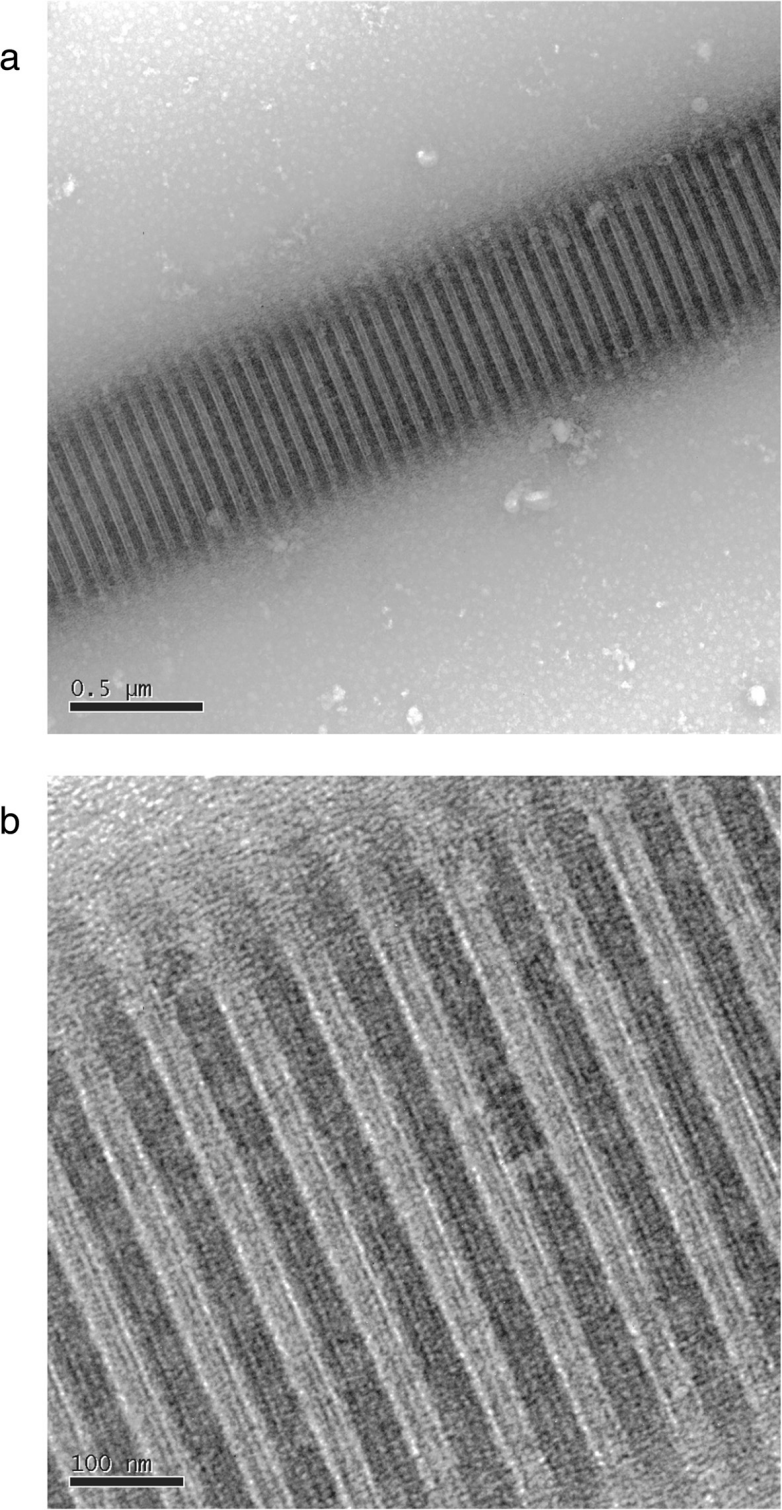
Transmission electron microscopy (TEM) shows evidence of highly ordered collagen fibrils. TEM images showing a section of a fibril formed in vitro from Lys-C-treated recombinant type II procollagen, exhibiting the dark/light D-banding pattern of a well-ordered structure, and showing substructure within each D period. Fibrils were negatively stained using 3 % uranyl acetate. **a** Scale bar = 500 nm. **b** Scale bar = 100 nm

As a final assay of fibril morphology and organization, we imaged fibrils formed from our recombinant type II collagen using transmission electron microscopy (TEM) (Fig. 9). TEM images show fibrils displaying distinct light/dark D-periodic banding patterns, a distinguishing feature of well-ordered collagen fibrils. Fibrils imaged using TEM consistently exhibited larger diameters than those formed for the AFM imaging experiments. We attribute this to the different protocols followed to initiate fibril formation in the two sets of experiments. It is well known that fibril properties can be influenced strongly by the conditions used for their formation [69]. Importantly, here the D-banding revealed in the TEM images confirms the formation of well-ordered fibrils, and the measured D-band spacing (69 nm) is consistent with literature values for type II collagen [70, 71]. This result offers a final demonstration of the native-like performance of our recombinantly expressed procollagen.

## Conclusions

Utilizing a human fibrosarcoma cell line, we have developed a recombinant system for expressing human type II procollagen. Demonstrated advances of this system over past approaches are (1) an easy-to-screen, non-covalently linked fluorescence reporter for transfected cells; (2) a demonstrated suite of post-translational modifications including hydroxylation and glycosylation in the resultant purified protein; and (3) a full-length native procollagen sequence, whose wide range of biophysical properties characterized within this work all correspond to the expected values of the native protein. This system should enable future work investigating the effects of chemical composition on these properties, and could provide a viable alternative to other approaches seeking to produce correctly modified collagen for materials or medical investigations.

## Methods

### Stable cell line construction

The human *COL2A1* gene was amplified from IMAGE consortium [41] CloneID 7486698 using primers that introduced a 5’ BglII restriction site and a 3’ BamHI site flanking the gene (forward primer: TTA GAG ATC TAC CAT GAT TCG CCT CGG GGC TCC CCA GAC GCT GG; reverse primer: TAA TCG GAT CCT ATT ACA AGA AGC AGA CCG GCC C). This allowed the direct replacement of the *EYFP* gene from pYIC (Addgene plasmid 18673) with the *COL2A1* gene, leaving an intact internal ribosomal entry site (IRES) to provide co-translational expression of ECFP in mammalian cells. DNA sequencing was used to verify the plasmid-based construct prior to transfection.

The construct was transfected into human fibrosarcoma cells (HT1080), [72] which were cultured at 37 °C with 8 % CO_2_ in Dulbecco’s modified Eagles media (DMEM, Mediatech) supplemented with 10 % foetal bovine serum (FBS, Invitrogen), 0.5 mM ascorbic-2-phosphate (Asc-2-P, Sigma) [73] and 20 mM 4-(2-Hydroxyethyl) piperazine-1-ethanesulfonic acid, N-(2-Hydroxyethyl) piperazine-N′-(2-ethanesulfonic acid) pH 7.2 (HEPES, Sigma). Plasmids were introduced into the cells using Superfect reagent (Qiagen) as per manufacturer’s recommendation [74]. Stable transformants were selected by the addition of geneticin (G418, Mediatech) to 400 μg/ml in the above media. Surviving cells were visually examined for ECFP fluorescence and selected clones expanded to confluence in T175 flasks (Sarstadt). Procollagen was precipitated from the clarified (5,000 g, 15 min) supernatant by the dropwise addition of 50 % aqueous PEG 3350 (Sigma) to a final concentration of 5 %, then harvesting by centrifugation (15,000 g, 15 min). The pellet was resuspended in a minimum volume of 100 mM Tris–HCl (Invitrogen) containing 403 mM NaCl (Caledon) and 25 mM EDTA (Bioshop) at pH 8.0. Select lines confirmed to be producing procollagen via Western blotting were re-cloned to homogeneity, expanded, and then stored in liquid nitrogen.

### Procollagen II purification

Secreted type II procollagen was purified from the stable cell line following a modification of a published protocol [17]. The procollagen producing cell line 2D12 was expanded to confluence into 1400 cm^2^ roller bottles under the conditions specified above, except with 0.2 mM Acs-2-P and 330 μg/ml G418. Once confluence was achieved, the media was replaced with harvest media (DMEM with 0.2 mM Asc-2-P and 20 mM HEPES pH 7.4). Media was subsequently removed by aspiration and replaced every 48 to 72 h for up to 2 weeks. Approximately 100 ml of 1 M Tris–HCl with 4 M NaCl and 250 mM EDTA pH 8.0 were added to each litre of media immediately after harvest, followed by clarification (5,000 g, 15 min, 4 °C). All subsequent steps were performed at 4 °C.

Proteins in the media were concentrated using either ammonium sulfate precipitation or tangential flow filtration. Ammonium sulfate ((NH_4_)_2_SO_4_, 175 mg/mL) was added to the clarified media to precipitate the procollagen overnight. The precipitate was harvested by centrifugation (7000 g for 4 h), the pellet was resuspended in 1X DE I buffer (50 mM Tris HCl, 100 mM NaCl, 2 mM EDTA, 1 M Urea, pH 7.4 at 4 °C), and the sample was further dialysed overnight against 1X DE I buffer to remove excess salts (4 °C, 12 kDa molecular weight cut-off). Alternatively, in a method that appeared to give a higher procollagen yield, the procollagen-containing media was concentrated from ~1 l to 50 ml using tangential flow filtration (Millipore Pellicon XL 100 kDa, 4 °C, ~13 h), and then dialysed as above into 1X DE I buffer. Following centrifugation (2,000 g for 10 min) to clarify the sample, it was passed through a diethylaminoethanol (DEAE) cellulose column (Sigma). The procollagen-containing flow-through was collected and immediately dialysed against several changes of Q Sepharose buffer (37 mM Tris–HCl, 1 mM EDTA, 1 M Urea, pH 8.5 at 4 °C). The dialysate was clarified as before, and then applied onto a Q Sepharose column (Sigma). The procollagen was eluted with a stepwise gradient of NaCl in Q Sepharose buffer. For long-term storage, the collagen was dialysed into 1X storage buffer (10 mM Tris HCl, 40 mM NaCl, 2.5 mM EDTA, pH 8.0) and kept at 4 °C.

Concentrations of collagen were determined using a Sircol-type assay, [75] using as a dye Sirius Red F3B (Direct Red 80, Sigma). The assay was validated by comparison with a commercial Sircol assay (Biocolor, with rat tail tendon collagen I as a standard) and using chicken sternal cartilage collagen (Sigma C9301) as a standard.

### Gel electrophoresis and Western blotting

Samples were run in 6 % polyacrylamide (Biorad) gels under reducing, denaturing conditions. Staining was performed with Coomassie blue (1 g Coomassie R250 in 40 % methanol) or silver (Biorad; volume used was half of manufacturer’s recommendation). Gels containing collagen fluorescently labelled with Alexa 647 (see below) were imaged with a gel scanner (Typhoon 9410 Gel and Blot Imager). For Western blotting, samples were transferred to 0.22 μm PVDF membranes (Biorad) and probed for the presence of procollagen with a collagen II specific monoclonal antibody (5B2.5, Abcam), which recognizes the sequence GGFDEK in the N-terminal telopeptide.

### Fluorescent labelling

Procollagen was labelled [76] with Alexa Fluor 647 carboyxlic acid, succinimidyl ester (Invitrogen A-20006) in 0.2 M carbonate-bicarbonate buffer pH 9.3 with 1 M NaCl, for 1 h at room temperature with gentle shaking in the dark. Unreacted fluorophores were removed using an HR-300 Sephadex column.

### MS/MS identification of tryptic fragments

Measurements were conducted at the UVic-Genome BC Proteomics Centre. Protein identity was established by searching against the Uniprot-Swissprot 20090225 (410518 sequences; 148080998 residues) all species, with the search parameters set to include modifications including hydroxyproline, hydroxylysine, glucosylgalactosyl hydroxylysine and galactosyl hydroxylysine, known post-translational modifications of collagen [77].

### Protease digestion

Chymotrypsin cleavage: Procollagen was digested with variable concentrations of chymotrypsin (Sigma, C7762) in 1X storage buffer, in volumes of 20 μl for 30 min at 4 °C. Reactions were stopped by adding 5 μl gel loading buffer.

Lys-C cleavage: Procollagen was incubated at 37 °C with lysyl endopeptidase (Lys-C, Roche, EC.3.4.21.50) [32] in 50 mM Tris buffer, pH 7.0 with 200 mM NaCl. Aliquots were removed at the specified time points and reactions were quenched by addition of an equal volume of protein gel loading buffer.

Cathepsin K cleavage: Recombinant procollagen was first digested by chymotrypsin to remove propeptides and generate type II collagen. Collagen was purified away from digested propeptides and chymotrypsin (spin filter, MWCO 50 kDA) and transferred into 1X activity buffer (100 mM sodium acetate, 2.5 mM EDTA, 2.5 mM dithiothreitol (DTT), pH 5.5). Digestions were performed at 28 °C in 1X activity buffer at concentrations of 0.6 mg/ml collagen, 400 nM of recombinant human cathepsin K [78] and 200 mM chondroitin sulfate (CSA) (Sigma-Aldrich). At the desired time points, aliquots were removed and inactivated for 30 min at room temperature with E64 (Sigma-Aldrich), a general cysteine protease inhibitor. Western blots were performed with an anti-type II collagen antibody cocktail (Chondrex, 7006).

### Circular dichroism (CD) spectroscopy

Following removal of propeptides via Lys-C digestion, collagen was exchanged into 0.2 M sodium phosphate for CD measurements. CD measurements were performed at 20 °C with a JASCO 810 CD spectrometer. The spectrum was measured at 0.5 nm wavelength increments and subsequently smoothed with a 10-point moving average. To determine a melting temperature, the ellipticity at 198 nm was monitored as the temperature was increased from 20 to 60 °C at a rate of 0.4 °C/min. The melting curve was fit with a sigmoidal expression

1 $$ E(T)={E}_2+\frac{\left({E}_1-{E}_2\right)}{\Big(1+ \exp \left(\frac{T-{T}_m}{dT}\right)} $$

to obtain an estimate for the melting temperature under these conditions. Here, *T*_m_ is the melting temperature, *dT* relates to the sharpness of the transition, and *E*_1_ and *E*_2_ represent the ellipticities before and after melting.

### Optical tweezers stretching

Single-molecule procollagen stretching experiments (Fig. 7a, inset) were performed using our home-built single-beam optical tweezers instrument [39, 79]. It uses a high numerical aperture objective lens (Olympus UPlanApo/IR, NA 1.2, 60 X water-immersions) to focus an 835 nm, 200 mW diode laser Gaussian beam into a flow chamber. A manually pulled glass micropipette is inserted in the flow chamber and mounted on a piezo-electric stage (Mad City Labs, Nano H-50), allowing it to be moved relative to the optical trap with nanometer-scale precision. The manipulation is in a plane perpendicular to the optical axis. Using a second, identical objective lens the laser light is re-collimated and directed onto a position sensitive photodiode (UDT Sensors, DL-10) that images the back-focal plane of the second objective. The photodiode detects deflections of the light as a result of the trapped object displacement from the trap center in directions perpendicular to the optical axis. In addition, images are recorded at 60 Hz using a CCD camera (Flea, Point Grey Research), from which the positions of the trapped and pipette-immobilized beads can be determined.

The deflection of the laser was used to determine the position of the trapped bead and its offset from equilibrium Δ*z*, and the stage read-out was used to determine the relative pipette bead position. These data were sampled at 1 kHz and were low-pass filtered to 10 Hz. Photodiode readings were calibrated based on positions of the trapped bead from video imaging. The video images are analyzed using an algorithm that fits a circle to the edge of the bead image (Labview 8.5, IMAQ Find Circular Edge), and were converted to distances in nanometers based on calibration of the camera.

To stretch single procollagen molecules, their ends were functionalized and bound to microspheres for manipulation. The cysteine residues in the globular propeptide ends were first reduced with beta-mercaptoethanol (Bioshop), and then were covalently biotinylated using maleimide-biotin (EZ-Link Maleimide-PEG2-Biotin, Thermo Scientific). Biotinylation was confirmed by Western blotting with streptavidin. The biotinylated procollagen was labeled with an antibody against the N-terminal propeptide (003-02, Abcam). The end-labeled procollagen sample was incubated with 2.1 μm diameter protein-G-coated polystyrene beads (Spherotech). In the optical tweezers instrument, the free biotinylated end of the molecule was attached specifically to a streptavidin-coated polystyrene bead held by suction on the tip of the micropipette [80]. This bead had a diameter of 1.27 μm, smaller than the trapped bead to be able distinguish it from the other bead and also to avoid optical interaction with the laser beam when a close separation from the trapped bead [79]. By moving the pipette, the end-to-end distance of the molecule was manipulated while positions of both beads were recorded. The resultant offset of the trapped bead Δ*z* reveals the force applied on the tethered molecule during manipulation. The relative separation of the two beads *z* gives the relative end-to-end distance of the molecule. Stretching experiments were performed in PBS buffer pH 7.4.

From the trapped bead displacement Δ*z* from equilibrium, force was calculated via *F* = -*κ*Δ*z*, where *κ* is the trap stiffness. Using power spectral analysis and fitting a Lorentzian to the data, trap stiffness is calculated from the resulting fitting parameter, corner frequency ${f}_c=\frac{\kappa }{2\pi \gamma }$ [81]. *γ* is the drag coefficient of the trapped particle, here assumed to be that corresponding to the nominal bead radius.

To analyze the response of the molecule to the applied force, the inextensible Worm-Like Chain (WLC) polymer elasticity model was used [82, 83].

2 $$ F(z)=\frac{k_BT}{p}\left[\frac{1}{4{\left(1-\frac{z}{L}\right)}^2}-\frac{1}{4}+\frac{z}{L}\right] $$

Here, *F*(z) provides the force required to achieve a given end-to-end extension of the molecule, *L* is the molecular contour length (300 nm for collagen [68]), *k*_*B*_ is Boltzmann’s constant, *T* is the absolute temperature, and *p* is the persistence length of the molecule. Because the positions of the beads are known only relatively and the exact binding point on the pipette bead is unknown, a length offset parameter, *o*, is added to equation (2):

3 $$ F(z) = \frac{k_BT}{p}\left[\frac{1}{4{\left(1-\frac{z-o}{L}\right)}^2}-\frac{1}{4}+\frac{z-o}{L}\right] $$

Force-extension curves were analyzed only if they ruptured to zero force in a single step, indicating tethering by a single molecule.

### Fibril formation

To make collagen fibrils for TEM analysis, purified procollagen was first dialyzed into phosphate buffered saline (PBS) pH 7.4 using Slide-A-Lyzer MINI Dialysis Units (20 kDa MWCO, Pierce) to a final concentration of 120 μg/ml. To initiate fibril formation, 25 μl of the concentrated procollagen was incubated with 3.5 μl of 10 μg/ml Lys-C (Roche) at 37 °C to remove N-and C-terminal propeptides [32]. Collagen fibrils formed after 24 h of incubation were isolated by centrifugation at 16,000 g for 45 min. The supernatant was removed and the pellet gently resuspended in 10 μl of PBS.

For AFM analysis, Lys-C cleavage of procollagen was followed by removal of enzyme and propeptide fragments by buffer exchange into 10 mM HCl via multiple passes through a Millipore YM-100 microcon filtration unit. This collagen was assembled into fibrils following the general approach of the “cold start” procedure [69]. The 100 μg/ml collagen monomer solution was mixed with phosphate buffer resulting in a solution of 0.05 M K_2_HPO_4_, 0.05 M KH_2_PO_4_ and 0.05 mg/ml collagen. The solution pH was adjusted to 7.0 by adding 0.01 M HCl or 0.01 M NaOH solutions. The sample was then incubated in a closed conical tube at 35 °C in a water bath. The solution pH remained around 7 throughout the experiment.

### Atomic force microscopy

Every 10 min, 10 μl solution was removed and diluted 100 times with ultrapure water (Barnstead, 18.2 MΩ · cm). Then 10–20 μl of diluted solution was deposited on a freshly cleaved mica surface. The sample was dried with a stream of dry, filtered compressed air for about 5 min and then mounted on the AFM stage for analysis.

All AFM experiments were performed using tapping mode (MikroMasch NSC35/CR-AU tips) under ambient conditions using an Asylum Research MFP-3D. Persistence lengths of the filaments were determined using the software 2D Single Molecules, as follows [84]. The filament contour was drawn by tracing along the filament direction. The program equally subdivided the contour curves into variable length vectors. These vectors were then analyzed to calculate persistence length *p* of the filament using the following equation assuming two-dimensional equilibration: [66]

4 $$ {\left\langle cos\theta \right\rangle}_{2D}= exp\left(-\frac{l}{2p}\right) $$

Here, *θ* is the angle between two tangent vectors separated by distance *l* along the filament contour. To extract the bending modulus, the following relation was applied, [85]

5 $$ p=\frac{\pi {E}_b}{64{k}_BT}\times {d}^4, $$

where *E*_*b*_ is the bending modulus of the filament and *d* is its diameter.

### Transmission electron microscopy

Samples were prepared by floating carbon-Formvar copper grids (300 mesh, Ted Pella) on 5 μl of resuspended collagen fibrils in PBS for 1 h. The grids were washed three times with deionized water, blotted with Whatman filter paper and then negatively stained with 2 % uranyl acetate (Ted Pella) for 45 s. Excess stain was removed by blotting and the grids were allowed to air dry at room temperature. The negatively stained samples were imaged at 200 kV accelerating voltage with a Hitachi 8000 transmission electron microscope at Simon Fraser University’s NanoImaging facility in 4D Labs.

## Additional file

Additional file 1: Figure S1. Results of mass spectrometric analysis on the recombinant human type II procollagen, demonstrating protein sequence coverage of COL2A1 and expected posttranslational modifications. (PDF 70 kb)

## Abbreviations

AFM: atomic force microscopy
Asc-2-P: ascorbic-2-phosphate
CCD: charge-coupled device
CD: circular dichroism
cDNA: complementary DNA
CSA: chondroitin sulfate
DEAE: diethylaminoethanol
DMEM: Dulbecco’s modified Eagles media
DTT: dithiothreitol
ECFP: enhanced cyan fluorescent protein
EDTA: ethylenediaminetetraacetic acid
EYFP: enhanced yellow fluorescent
FBS: foetal bovine serum
HEPES: 4-(2-hydroxyethyl)-1-piperazineethanesulfonic acid
IRES: internal ribosomal entry site
MOWSE: MOlecular Weight SEarch
MS/MS: tandem mass spectrometry
MW: molecular weight
MWCO: molecular weight cut-off
PBS: phosphate buffered saline
PEG: polyethylene glycol
TEM: transmission electron microscopy
*T*_m_: melting temperature
WLC: worm-like chain
