# Supplementary material for: Development and characterization of a eukaryotic expression system for human type II procollagen
Source: BMC Biotechnol. 2015 Dec 15;15:112. doi: 10.1186/s12896-015-0228-7 (PMC4678704; doi:10.1186/s12896-015-0228-7)
Supplement: Additional file 1: Figure S1. — Results of mass spectrometric analysis on the recombinant human type II procollagen, demonstrating protein sequence coverage of COL2A1 and expected posttranslational modifications. (PDF 70 kb) [file 12896_2015_228_MOESM1_ESM.pdf]

Development and characterization of a eukaryotic expression system for human type II procollagen

Andrew Wieczorek, Naghmeh Rezaei, Clara K. Chan, Chuan Xu, Preety Panwar, Dieter Brömme, Erika F. Merschrod S. and Nancy R. Forde*

Supporting Information

(a)

1 MIRLGAPQTL VLLTLLVAAV LRCQGQDVQE AGSCVQDGQR **YNDKDVWKPE**
51 **PCR**ICVCDTG TVLCDDIICE DVKDCLSPEI PFGECCPICP TDLATASGQP
101 GPKGQKGEPG DIKDIVGPK**G PPGPQGPAGE QGPR**GDRGDK GEKGAPGPRG
151 RDGEPGTPGN PGPPGPPGPP GPPGLGGNFA AQMAGGFDEK **AGGAQLGVMQ**
201 **GPMGPMGPRG PPGPAGAPGP QGFQGNPGEP GEPGVSGPMG PRGPPGPPGK**
251 **PGDDGEAGKP GK**AGER**GPPG PQGARGFPGT PGLPGVKGHR** GYPGLDGAKG
301 EAGAPGVKGE SGSPGENGSP GPMGPRGLPG ERGR**TGPAGA AGARGNDGQP**
351 **GPAGPPGPVG PAGGPGFPGA PGAK**GEAGPT GAR**GPEGAQG PRGEPGTPGS**
401 **PGPAGASGNP GTDGIPGAKG SAGAPGIAGA PGFPGPRGPP GPQGATGPLG**
451 **PK**GQTGEPGI AGFKGEQGPK GEPGPAGPQG APGPAGEEGK RGAR**GEPGGV**
501 **GPIGPPGER**G APGNR**GFPGQ DGLAGPKGAP GERGPSGLAG PK**GANGDPGR
551 PGEPGLPGAR **GLTGRPGDAG PQGKVGPSGA PGEDGRPGPP GPQGARGQPG**
601 **VMGFPGPK**GA NGEPGK**AGEK GLPGAPGLRG LPGKDGETGA AGPPGPAGPA**
651 **GERGEQGAPG PSGFQGLPGP PGPPGEGGKP GDQGVPGEAG APGLVGPR**GE
701 RGFPGER**GSP GAQGLQGPRG LPGTPGTDGP KGASGPAGPP GAQGPPGLQG**
751 **MPGERGAAGI AGPKGDRGDV GEKGPEGAPG KDGGRGLTGP IGPPGPAGAN**
801 **GEKGEVGPPG PAGSAGAR**GA PGERGETGPP GPAGFAGPPG ADGQPGAKGE
851 QGEAGQKGDA GAPGPQGPSG APGPQGPTGV TGPKGAR**GAQ GPPGATGFPG**
901 **AAGR**VGPPGS NGNPGPPGPP GPSGKDGPKG ARGDSGPPGR **AGEPGLQGPA**
951 **GPPGEKGEPG DDGPSGAEGP PGPQGLAGQR GIVGLPGQR**G ER**GFPGLPGP**
1001 **SGEPGKQGAP GASGDRGPPG PVGPPGLTGP AGEPGREGSP GADGPPGRDG**
1051 **AAGVKGDRGE TGAVGAPGAP GPPGSPGPAG PTGKQGDRGE AGAQGPMGPS**
1101 **GPAGARGIQG PQGPRGDKGE AGEPGER**GLK GHR**GFTGLQG LPGPPGPSGD**
1151 **QGASGPAGPS GPR**GPPGPVG PSGK**DGANGI PGPIGPPGPR** GR**SGETGPAG**
1201 **PPGNPGPPGP PGPPGPGIDM SAFAGLGPRE KGPDPLQYMR ADQAAGGLRQ**
1251 **HDAEVDATLK SLNNQIESIR** SPEGSRKNPA RTCRDLK**LCH PEWKSGDYWI**
1301 **DPNQGCTLDA MKVFCNMETG ETCVYPNPAN VPKKNWWSSK** SKEKKHIWFG
1351 ETINGGFHFS YGDDNLAPNT ANVQMTFLRL LSTEGSQNIT YHCK**NSIAYL**
1401 **DEAAGNLKKA LLIQGSNDVE IR**AEGNSR**FT YTALK**DGCTK HTGKWGKTVI
1451 EYRSQKTSRL PIIDIAPMDI GGPEQEFGVD IGPVCFL

(b)

Start	End	Peptide sequence, indicating posttranslationally modified sites (underlined)	Hydroxylation	Glycosylation
516	527	GFPGQDGLAGPK	Proline (1)<br>Lysine (1)	
516	533	GFPGQDGLAGPKGAPGER	Proline (2)	Galactosyl-hydroxylysine
516	533	GFPGQDGLAGPKGAPGER	Proline (2)	Glucosyl-galactosyl-hydroxylysine
1193	1229	SGETGPAGPPGNPGPPGPPGPPGPGIDMSAFA GLGPR	Proline (5)	
1193	1229	SGETGPAGPPGNPGPPGPPGPPGPGIDMSAFA GLGPR	Proline (6)	
1193	1229	SGETGPAGPPGNPGPPGPPGPPGPGIDMSAFA GLGPR	Proline (7)	

Figure S1. Results of LC-MS/MS analysis on our recombinant human type II procollagen. (a) Protein sequence coverage of the COL2A1 protein from peptides identified in LC-MS/MS following trypsin digestion of protein chains. The overall sequence coverage from this analysis was 62%, with COL2A1 the top-scoring match identified from the Uniprot-Swissprot database (410518 sequences). Bold red indicates peptide matches. (b) Two examples of peptides identified that occur with variable posttranslational modifications.
